# Lingual Raynaud's Phenomenon after Surgical and Radiotherapeutic Intervention for Oral Squamous Cell Carcinoma

**DOI:** 10.1155/2022/1567581

**Published:** 2022-08-19

**Authors:** Nicholas J. Murphy, Loay S. Kabbani, Alexander D. Shepard, Farzan Siddiqui

**Affiliations:** ^1^Wayne State University School of Medicine, 540 E. Canfield Ave. Detroit, MI 48201, USA; ^2^Department of Surgery, Henry Ford Hospital, 2799 W Grand Blvd Detroit, MI 48202, USA; ^3^Department of Radiation Oncology, Henry Ford Hospital, 2799 W Grand Blvd Detroit, MI 48202, USA

## Abstract

Raynaud's phenomenon of the tongue after radiation therapy with or without chemotherapy is an exceedingly rare complication. Symptoms are similar to Raynaud's disease of other sites and involve pallor and discomfort on exposure to cold temperatures that resolve with rewarming. Presentation occurs approximately 18-24 months after radiotherapy on average and can usually be managed effectively with lifestyle modification and pharmacotherapy. Here, we present a case of lingual Raynaud's following surgery and adjuvant radiation therapy in a patient with squamous cell carcinoma of the oral cavity.

## 1. Introduction

Raynaud's phenomenon (RP) is a vasospastic process that typically affects the digits of the hands, causing painful discoloration on exposure to cold temperatures or strong emotions [1]. It is estimated that 3-5% of the general population is affected by RP with a predilection for females and those in more northerly regions [1]. Classically, it presents in three phases beginning with pallor, which represents the initial vasoconstrictive ischemic phase. After a prolonged period of ischemia, the second phase of a cyanotic blue color results from the presence of desaturated blood in the affected part. With recovery, there is a final hyperemic phase of vasodilation which produces a red color. This classic color progression from white to blue to red is only infrequently present. Episodes of vasospasm usually last from minutes to one hour but can last multiple hours [1].

The precise mechanism for RP has not yet been elucidated but current thinking points to a combination of probable neural and vascular factors. The neural pathway involves sympathetic nervous system stimulation by cold temperatures or stress. The resulting release of norepinephrine activates vascular smooth muscle alpha adrenoreceptors leading to vasoconstriction. However, local factors can override this neural response as seen in patients with local anesthesia of sympathetic nerves or postsympathectomy who can still experience episodes of RP [1]. Another postulated mechanism for RP is production of reactive oxygen species (ROS) by mitochondria in response to cold temperatures [1]. Signaling through the Rho/Rho-kinase pathway leads to translocation and subsequent activation of *α*2C-adrenoreceptors causing vasoconstriction [1].

Small artery occlusive disease (e.g., vasculitis) is more commonly implicated in secondary RP, which is associated with collagen vascular diseases such as systemic sclerosis [1]. Some of the vascular causes include endothelial cell damage, disparate vasoactive substance production, intravascular narrowing, and increasing vasoconstriction [1]. Once exposed to cold, these factors increase the body's natural vasoconstrictive response leading to the classical pale skin of affected areas [1, 2].

Development of management strategies has been hampered by the complex etiology of RP and a poor understanding of its exact mechanisms. Many therapies have been tried with varying degrees of success. They are broadly divided into nonpharmacologic and pharmacological interventions.

Treatment is stratified based on severity, beginning with nonpharmacological approaches for mild symptoms. These include lifestyle modification to avoid triggers such as keeping the affected areas warm with clothing and avoiding exposure to cold temperatures [1]. Smoking cessation is always advised, if applicable [1]. For more severe symptoms, vasodilator therapy is added, typically beginning with dihydropyridine calcium channel blockers—nifedipine being the most studied and drug of choice [1]. Other potential agents include alpha-1 adrenoreceptor antagonists (prazosin), angiotensin-converting enzyme inhibitors, nitric oxide, prostaglandin analogs, phosphodiesterase inhibitors, selective serotonin reuptake inhibitors, antioxidants, botulinum toxin A, endothelin receptor antagonists, and Rho inhibitors [1]. Alternative approaches include herbal remedies such as Ginkgo biloba extract and rosemary oil, acupuncture, transcutaneous nerve stimulation, and sympathectomy among others [1].

Although the primary regions affected by RP are the digits of the hands and feet, rarely affected regions include the nose, ears, lips, and nipples. Raynaud's of the tongue is exceedingly rare and to date is only described in a handful of case reports.

## 2. Case

**Figure 1:**
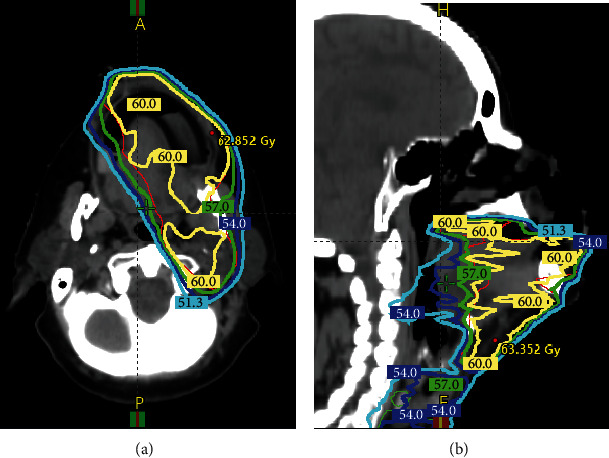
(a, b) Dose lines of radiotherapy treatment showing irradiated regions of the oral cavity.

**Figure 2:**
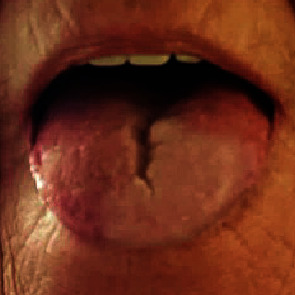
Picture of the oral tongue demonstrating left-sided tongue pallor.

A 63-year-old postmenopausal female with a history of hypertension, hyperlipidemia, and former 25 pack-year tobacco use presented with squamous cell carcinoma of the oral cavity pathological stage IVA (pT1N2bM0). She had undergone wide local excision of an alveolar ridge lesion with, left neck dissection of levels I-IV, and reconstruction with a right radial forearm free flap; the margins were negative, and there was no extranodal extension. She subsequently completed postoperative radiation therapy to a dose of 60 Gy in 30 fractions using intensity-modulated radiation therapy (IMRT) to the areas shown in Figures 1(a) and 1(b). At follow-up, 19 months after radiation, she continued to suffer from xerostomia and diminished taste sensation but showed no evidence of disease based on clinical and radiologic examination. Approximately two years after completion of radiation, in the month of October, she began to notice intermittent lingual pallor. She reported that the tip and anterior 1/4 of her tongue predominately on the left side began to tingle and turn white, lasting 10-15 minutes and occurring two to three times per day as shown in Figure 2. This change occurred mostly with exposure to cold ambient temperatures and resolved with rewarming. Interestingly, it did not occur with drinking cold beverages. There were no motor, sensory, balance, or hearing changes or similar symptoms in the digits of the hands or feet. She had no history of Raynaud's disease, cerebrovascular accident, transient ischemic attack, or other neurologic disease. Physical examination revealed no visible abnormality of the tongue and flexible laryngoscopy was unremarkable. A computed tomography angiogram of the head and neck done to investigate possible vascular causes showed no significant extracranial arterial occlusive disease or aneurysm. She was referred to vascular surgery for further evaluation, and laboratory studies were ordered, showing an ESR 4 mm/Hr, CRP 0.2 mg/dL, PT 12.9 sec, PTT 25 sec, INR 0.97, DRVVT 35 sec, and lupus anticoagulant negative. Her presentation was noted to be most consistent with Raynaud's phenomenon of the tongue, and a trial of nifedipine extended release 30 mg daily was started. During follow-up several weeks later, she reported significant improvement in the frequency and severity of symptoms and was tolerating treatment, though attacks continued to occur intermittently when outdoors in cold temperatures.

## 3. Discussion

Raynaud's phenomenon of the tongue is exceedingly uncommon and often occurs in the setting of primary Raynaud's disease of the digits. There have been at least 22 cases of lingual involvement reported in the literature, usually associated with systemic sclerosis, Sjögren's syndrome, or other connective tissue disorders [3–8]. Of the lingual Raynaud's cases found in the literature, four were identified without preexisting connective tissue disease all occurring after the use of radiotherapy for head and neck squamous cell carcinomas [2, 9–11]. In three cases, there was no history of Raynaud's disease affecting the digits and in all cases, radiotherapy had occurred between 18 and 54 months prior to the onset of symptoms. One of the patients was noted to have a smoking history while the other three were either nonsmokers or not specified. Nicotine is noted to be a risk factor for Raynaud's disease and was potentially a contributing factor in our patient [1].

Interestingly, 2/4 cases involved male patients, while typical RP tends to affect females more than males. One risk factor for RP is the hormone estrogen, which potentiates vasoconstriction occurring via alpha adrenoreceptors [1]. Further evidence for this factor comes from estrogen therapy increasing the risk of RP in postmenopausal women [1]. Our patient was not on estrogen therapy. Raynaud's phenomenon can result from either increased vasoconstriction or impaired vasodilation or a combination of the two in small arteries. Underlying arterial occlusive disease can be a major contributing factor. Arteries narrowed by such a process have a lower “critical closing pressure,” and normal cold-induced vasoconstriction can lead to temporary vessel closure. Radiation leads to a progressive obliterative endarteritis of small vessels which makes them prone to such changes and may be a contributing factor to the rare occurrence of RP [2]. Additionally, radiation damage to the endothelium disrupts normal chemical mediators of vasorelaxation. The result is exaggerated vasoconstriction, both in intensity and duration, to normal adrenergic-mediated stimuli (e.g., cold or stress) and impaired vasodilation leading to the classic symptoms of RP [2]. Surgery is also likely a contributing factor from scarring leading to compression of small arterial beds [11].

Non-RT-associated cases in this group of lingual Raynaud's patients tended to occur in younger people and females, often with a previously diagnosed systemic sclerosis with digital Raynaud's symptoms [3–8].

Treatment of our patient using calcium channel blockers was apparently effective, though other options ranging from lifestyle changes of avoiding colder temperatures up to recurrent botulinum toxin injections seemed acceptable [2, 9–11]. Three of the other cases demonstrated stable disease with nonpharmacologic management, leading to ongoing intermittent pain attacks that were reported as self-limiting and tolerable [2, 9, 10]. One patient was treated with fifty units of botulinum toxin injected along the sternocleidomastoid muscle, which provided complete resolution of the patient's discoloration, paresthesia, and dysphagia for 3 months until symptoms gradually returned [11]. Repeat injections reportedly had similar effect to the first [11]. Overall, the treatment paradigm for lingual Raynaud's after RT seems to mirror the general consensus for primary Raynaud's disease. Severity of symptoms and patient preference for intervention should be the main considerations until further data in this specific patient population become available.

## 4. Conclusion

In summary, lingual Raynaud's phenomenon is a rare complication of radiation therapy to the oral cavity. Although the exact mechanism is currently unknown, the etiology is likely related to radiation-induced small artery damage resulting in an exaggerated vasoconstrictive response to normal adrenergic stimulation. Treatments can be effective in mitigating symptoms and include lifestyle changes and pharmacologic intervention.

## Data Availability

The cases discussed in this report may be accessed at the provided references. There is no additional unreleased data.
